# Metabolomic Signatures of Transitional Breast Milk in Gestational Diabetes Mellitus: A Case–Control Study Assessing the Impact of Insulin Therapy

**DOI:** 10.3390/nu17193101

**Published:** 2025-09-29

**Authors:** Merve Küçükoğlu Keser, Sıddika Songül Yalçin, Tuba Reçber, Emirhan Nemutlu

**Affiliations:** 1 Department of Pediatrics, Ankara City Hospital, Ankara 06800, Türkiye; 2 Department of Social Pediatrics, Institute of Child Health, Hacettepe University, Ankara 06230, Türkiye; siyalcin@hacettepe.edu.tr; 3 Department of Analytical Chemistry, Faculty of Pharmacy, Hacettepe University, Ankara 06100, Türkiye; tuba.recber@hacettepe.edu.tr (T.R.); enemutlu@hacettepe.edu.tr (E.N.)

**Keywords:** Gas Chromatography-Mass Spectrometry, gestational diabetes, human milk, insulin, metabolomic

## Abstract

**Background/Objectives**: Gestational diabetes mellitus (GDM) alters maternal metabolism during pregnancy and may impact the biochemical composition of breast milk. Given the critical role of human milk in early-life metabolic programming, identifying metabolic alterations in GDM milk and understanding the effects of insulin therapy has important implications for neonatal health. This study aims to investigate the metabolomic profile of transitional breast milk in mothers with gestational diabetes mellitus compared with healthy controls and to evaluate the impact of insulin therapy on milk metabolite composition. **Methods**: Breast milk samples were collected between postpartum days 10 and 15 from 80 mothers with GDM and 80 matched controls. Metabolomic profiling was performed using gas chromatography–mass spectrometry (GC–MS), and data were analyzed using multivariate and univariate statistical techniques including PCA, PLS–DA, logistic regression, and ROC analysis. **Conclusions**: A total of 133 metabolites were identified, and GDM mothers exhibited a distinct metabolomic signature characterized by significant alterations in carbohydrate, amino acid, and microbial-derived metabolites. In particular, galactinol, arabitol, and pyrogallol were significantly decreased, while α-ketoglutaric acid and citric acid were elevated in the GDM group. Insulin-treated mothers showed unique metabolic changes involving glycolytic intermediates (glycerone phosphoric acid), purine metabolism (xanthine), and oxidative pathways (isocitric acid, gluconic acid lactone). Multivariate models based on the top metabolites achieved moderate discriminatory performance (AUC = 0.68). GDM is associated with substantial metabolic changes in transitional breast milk, and insulin therapy appears to modulate these alterations further. These findings suggest that maternal metabolic status and its treatment can shape the neonatal nutritional environment, potentially influencing early metabolic programming.

## 1. Introduction

Gestational diabetes mellitus (GDM) is one of the most common metabolic complications of pregnancy, affecting an estimated 5–20% of pregnancies worldwide and rising in prevalence [1,2]. GDM is characterized by hyperglycemia due to insufficient insulin secretion to meet pregnancy-induced insulin resistance [3,4]. This condition carries significant short- and long-term health risks. If untreated, it can lead to adverse perinatal outcomes (e.g., preeclampsia, birth trauma, neonatal hypoglycemia) [5,6]. Additionally, GDM predisposes mothers to future type 2 diabetes and cardiovascular disease [7,8], while offspring have a higher risk of early-onset obesity and metabolic syndrome [9,10]. Effective management of GDM (through diet, exercise, and/or insulin therapy) is critical to reduce these risks [2,11]. However, even with treatment, subtle metabolic disturbances may persist and could influence the breast milk composition that nourishes the infant [12,13].

Early-life nutrition plays a pivotal role in metabolic programming. Human breast milk is not only a source of calories but also contains numerous bioactive metabolites that can impact infant growth, immunity, and metabolism [12]. Understanding how GDM affects breast milk’s metabolome is important, given that infants of GDM mothers have an elevated risk of obesity and glucose intolerance later in life [9,10]. Metabolomics offers a powerful approach to uncover such changes, as it provides an integrated snapshot of metabolic pathways [14]. Prior omics research has shown that multifactorial diseases like GDM require comprehensive analysis of metabolites and pathways rather than single biomarkers [15,16]. Technological advances now enable high-throughput profiling of biofluids, and metabolomic analyses can sensitively detect disease-associated metabolic shifts [17]. Indeed, metabolomics has revealed significant alterations in maternal biofluids (plasma, urine, etc.) in GDM [15]. For example, studies of maternal blood have identified early pregnancy differences in amino acids and lipids that predict GDM [16,18]. However, studies specifically examining the human milk metabolome in GDM remain limited [12].

Emerging research indicates that GDM can alter breast milk composition [19,20,21]. Wu et al. analyzed transitional milk and found 30 metabolites (mostly glycerophospholipids and fatty acids) significantly lower and 19 metabolites (including amino acids and nucleotides) higher in GDM mothers’ milk compared to controls [19]. Wen et al. evaluated colostrum, transitional, and mature milk, identifying significant GDM-related differences in 28 metabolites, particularly changes in lipids and amino acids over lactation stages [20]. Yao et al. analyzed mature milk and reported that GDM was associated with elevated fatty acids (linoleic and arachidonic acids) and related oxidized metabolites (e.g., 9R-HODE), as well as increased L-glutamic acid and reduced 12,13-DHOME [21]. These prior studies suggest that GDM consistently affects certain metabolic pathways in milk (e.g., lipid and carbohydrate metabolism), but they did not examine whether maternal insulin treatment modifies these effects [19,20,21]. Insulin therapy is used in GDM when lifestyle measures are insufficient, and it has systemic metabolic effects that could plausibly influence milk composition. Notably, there is a paucity of data on how pharmacological insulin intervention in GDM mothers may modulate the milk metabolome.

To date, no study has specifically stratified GDM mothers by insulin use to investigate differences in milk metabolites. It remains unclear whether mothers with insulin-treated GDM have a milk metabolic profile distinct from those managed with diet alone. Addressing this gap is important because any insulin-related changes in milk constituents could have downstream implications for the infant’s metabolism. Therefore, in this case–control study we aimed to characterize the metabolomic signatures of transitional breast milk (postpartum days 10–15) in GDM mothers versus healthy controls and to determine whether insulin therapy for GDM further alters the milk metabolome. We employed untargeted GC–MS-based metabolomics to comprehensively profile milk metabolites and performed subgroup analyses between insulin-treated and non-insulin GDM. We hypothesized that GDM would be associated with distinct milk metabolite patterns relative to controls and that insulin treatment would modulate those patterns. Our findings are intended to augment the limited literature in this field and provide novel insight into how maternal metabolic interventions might shape the nutritional and bioactive milieu of breast milk.

## 2. Materials and Methods

### 2.1. Study Design and Setting

This prospective case–control study was conducted between January and June 2023 at the Neonatal Outpatient Clinic of Ankara Bilkent City Hospital, a tertiary care facility in Türkiye. The primary aim was to investigate the metabolomic composition of transitional breast milk in mothers diagnosed with GDM, with a specific focus on the impact of insulin therapy.

The study was approved by the institutional ethics committee (Number E2-23-3916), and written informed consent was obtained from all participants.

### 2.2. Study Population

Eligible participants included mothers aged 18–49 years with a diagnosis of GDM, who presented voluntarily for routine neonatal follow-up. The diagnosis of GDM was based on the International Association of Diabetes and Pregnancy Study Groups (IADPSG) criteria.

For each GDM participant, a mother without GDM or any known chronic illness was recruited as a control. The mothers in both groups had term, singleton pregnancies and typically healthy newborns [22].

Exclusion criteria included mothers who were not breastfeeding or who refused to provide breast milk, those unable to provide ≥5 mL milk, mothers with pre-existing type 1 or type 2 diabetes or other chronic metabolic diseases, multiple gestations, and use of systemic medications within five days prior to milk collection. In particular, recent antibiotic or analgesic use around the time of sample collection led to exclusion, to avoid confounding effects of those medications on milk composition. (All participants reported no antibiotic or pain medication use in the week prior to sampling.) We relied on self-reporting for medication/supplement use; although we carefully queried mothers, we acknowledge that self-reported intake may be imperfect.

Within the GDM group (*n* = 80), participants were further stratified by insulin therapy status (insulin-treated *n* = 49; diet-controlled *n* = 31) for subgroup comparisons. A post hoc power analysis confirmed that our total sample size of 160 (80 GDM, 80 controls) exceeded the requirement (128 total for 80% power to detect medium effect size d = 0.5 at α = 0.05), indicating adequate power to detect moderate metabolite differences between groups.

### 2.3. Study Procedures

#### 2.3.1. Visit 1 (Postpartum Days 2–3)

During the first postpartum hospital visit, participants received detailed information about the study and provided written consent. A structured questionnaire was used to collect sociodemographic characteristics, pre-pregnancy weight, gestational weight gain, prenatal care history, and delivery details.

Medical records were reviewed to obtain relevant laboratory results, including fasting plasma glucose and oral glucose tolerance test results.

All the mothers received standardized breastfeeding counseling and training on manual milk expression techniques in preparation for the follow-up visit.

#### 2.3.2. Visit 2 (Postpartum Days 10–15)

At the second visit, the mothers were asked about their breastfeeding status and any difficulties encountered. To minimize variability due to colostrum composition and postpartum medication use, transitional breast milk samples were collected between postpartum days 10 and 15.

Transitional breast milk samples were collected from all participants between postpartum days 10 and 15, following a standardized protocol. The mothers were instructed to collect the samples in the morning between 09:00 and 11:00 after at least 2 h without breastfeeding or pumping. Milk was expressed manually from the same breast after nipple cleansing with sterile water. A midstream portion of approximately 5 mL was collected into pre-labeled sterile polypropylene tubes. Samples were immediately placed on ice and transferred to the laboratory within 30 min. Upon arrival, they were stored at −80 °C until analysis.

### 2.4. Breast Milk Sample Preparation

To prepare samples for metabolomic analyses, 200 μL of breast milk was sequentially mixed with 600 μL methanol, 200 μL water, and 600 μL chloroform. After vortexing and centrifugation at 15,000 rpm for 10 min at 4 °C, the upper aqueous phase (400 μL) was collected for metabolomic profiling. Samples were evaporated to dryness under nitrogen and stored at −80 °C until further analysis. All solvents used were of MS-grade purity (Sigma-Aldrich, Burlington, MA, USA).

### 2.5. GC–MS-Based Metabolomic Analysis

Gas chromatography–mass spectrometry (GC–MS)-based metabolomic profiling was carried out following the protocol described in our previous studies [23]. In brief, the dried residues were methoximated with 20 µL of methoxyamine hydrochloride (20 mg/mL in pyridine) at 30 °C for 90 min. Subsequently, 80 µL of MSTFA containing 1% TMCS was added to the samples at ambient temperature, and derivatization was performed at 37 °C for 30 min.

In quantitative assays, internal standards are commonly used to correct for variability introduced during sample preparation, instrument performance, and matrix effects. However, this strategy is limited to a small number of metabolites. As in other untargeted metabolomics studies, where the primary goal is to profile as many metabolites as possible, it was not feasible to apply internal standards covering all compounds. Therefore, pooled quality control (QC) samples representing the entire dataset were employed for normalization and to minimize analytical variability.

After derivatization, the samples were transferred to silylated GC–MS vials and analyzed on a Shimadzu GC–MS QP2010 Ultra equipped with a DB-5MS column (30 m + 10 m DuraGuard × 0.25 mm i.d., 0.25 µm film). The solvent delay was set at 4 min. The oven temperature program began at 60 °C for 1 min, increased at 15 °C/min to 325 °C, and was held for 8 min. The MSD transfer line temperature was maintained at 250 °C, helium carrier gas flow at 1 mL/min, and the mass range was 50–650 Da. The total run time was 26.67 min.

To ensure data reliability, standardized procedures were implemented during breast milk collection, storage, and preparation to minimize technical variation. QC and blank samples were injected at regular intervals to monitor instrument performance and provide statistical control of precision and accuracy. The injection order of study samples was randomized to limit instrument-related drift. During data processing, a QC-based normalization approach was applied to correct for differences in sample load and signal fluctuation, thereby ensuring consistency and accuracy of the metabolomic data.

### 2.6. Data Preprocessing and Feature Extraction

Raw GC–MS data were processed using MS-DIAL v4.92 for deconvolution, alignment, and normalization. Metabolite identification was based on the Fiehn Retention Index Library with a similarity threshold of ≥70%. Data normalization included total ion current (TIC) correction and LOWESS smoothing.

Metabolites with >50% missing data were excluded. Remaining missing values were imputed as one-fifth of the lowest observed value. Log2 transformation and Pareto scaling were applied to stabilize variance and improve comparability between features.

### 2.7. Statistical and Multivariate Analysis

Descriptive statistics, including arithmetic mean, standard deviation, median, and percentage distributions, were used to summarize the data. Distribution normality was assessed using the Kolmogorov–Smirnov test. Group comparisons and associations were analyzed using the Student’s *t*-test, Chi-square test, and ANOVA, as appropriate. A *p*-value of <0.05 was considered statistically significant.

#### 2.7.1. Group Comparison Using Non-Parametric Mann–Whitney U Test

To compare metabolite levels between the GDM and control groups with skewed distribution, the non-parametric Mann–Whitney U test was employed.

#### 2.7.2. Data Analysis with R Software

All analyses were conducted in R software (version 4.5.1) using the following packages: limma, glmnet, randomForest, caret, pROC, ggplot2, factoextra, and ComplexHeatmap. Prior to any analysis, the dataset was appropriately preprocessed, and the analysis pipeline included exploratory data analysis, univariate and multivariate testing, biomarker selection, model building and validation, and biological interpretation through pathway enrichment analysis.

#### 2.7.3. Data Preprocessing

Before statistical analysis, raw metabolite concentrations were log2-transformed to stabilize variance and approximate normality. Subsequently, Pareto scaling (mean-centering followed by division by the square root of the standard deviation) was applied to reduce the impact of large-scale differences between metabolites while retaining biological variance. The dataset was transposed when required to match the expected input structure for certain functions (e.g., for limma), ensuring that rows represented samples and columns represented metabolites.

#### 2.7.4. Exploratory Data Analysis

Principal component analysis (PCA) was conducted using the factoextra package to visualize global patterns and detect potential outliers or batch effects in the dataset. PCA plots were inspected to evaluate the overall structure and variance explained by principal components. Samples lying outside the 95% confidence ellipse in PCA plots were considered potential outliers and examined for technical or biological reasons before exclusion.

#### 2.7.5. Partial Least Squares Discriminant Analysis (PLS–DA)

To assess discriminatory metabolomic signatures, a PLS–DA model was built using mixOmics or equivalent functions, incorporating log2-transformed and scaled data. Class labels (GDM vs. control) were used as the outcome variable. Metabolite importance was ranked using variable importance in projection (VIP) scores, with a threshold of VIP > 1 indicating significant contribution to class separation.

#### 2.7.6. Univariate Analysis Using Generalized Linear Models (GLMs)

To identify individual metabolites associated with GDM status, we performed univariate logistic regression analyses. Each metabolite was independently modeled as a predictor of GDM (binary outcome: GDM vs. control) using the GLM function with a binomial family. The odds ratio (OR) and *p*-value for the metabolite term were extracted. False Discovery Rate (FDR) adjustment of *p*-values was conducted using the Benjamini–Hochberg method to control for multiple testing. The top-ranking metabolites were reported based on FDR-adjusted *p*-values.

#### 2.7.7. Random Forest Classification

A Random Forest classifier was constructed using the Random Forest package. Feature importance was derived using the mean decrease in Gini index. The model was optimized using 5-fold cross-validation.

#### 2.7.8. LASSO Logistic Regression

To further select predictive features, LASSO logistic regression with L1 regularization was performed using the glmnet package. The regularization parameter (λ) was optimized via cross-validation, and features with non-zero coefficients were retained as predictive biomarkers.

#### 2.7.9. Biomarker Evaluation

Individual Receiver Operating Characteristic (ROC) analyses were conducted for metabolites with VIP > 2, using the pROC package. Additionally, multivariate logistic regression and Random Forest models were constructed using top-performing metabolites (e.g., galactinol, arabitol, α-ketoglutaric acid, *p*-cresol, pyrogallol). The Area Under the Curve (AUC) was calculated to assess model performance.

To ensure generalizability and prevent overfitting, 5-fold cross-validation was employed. The dataset was randomly partitioned into five equal parts, iteratively training on 80% and testing on 20%. The mean AUC across folds was reported as the overall model performance.

#### 2.7.10. Model Performance and Validation

Model performance was evaluated based on Confusion Matrix (using the caret package), Accuracy, Sensitivity, Specificity, Positive/Negative Predictive Values, Balanced Accuracy, Cohen’s Kappa, McNemar’s test.

#### 2.7.11. Covariate-Adjusted Analysis

To account for potential confounding variables, we conducted multivariate logistic regression analyses. Each metabolite was entered separately into a logistic regression model adjusting for maternal age, gestational duration, and BMI. The models were fitted using the GLM function with a binomial family, where GDM status served as the dependent variable. The odds ratio (OR) and *p*-value for each metabolite were extracted while controlling for the covariates. FDR-adjusted *p*-values (Benjamini–Hochberg correction) were calculated to account for multiple comparisons, and the most significant metabolites were identified accordingly.

## 3. Results

### 3.1. Mother/Newborn Characteristics

The demographic and clinical characteristics of the study participants are summarized in Table 1. Among the GDM mothers, 61.3% were receiving insulin therapy during pregnancy.

GDM, gestational diabetes mellitus; BMI, Body mass index; PP, postpartum; SGA, small for gestational age; AGA, appropriate for gestational age; LGA, large for gestational age.

### 3.2. Global Metabolomic Pattern: PCA and PLS–DA

**Figure 1:**
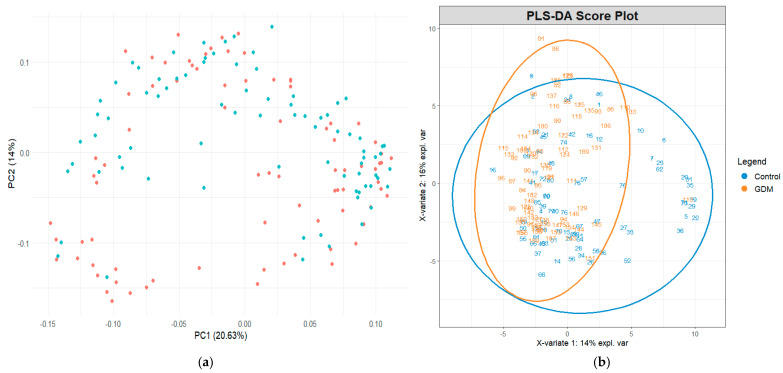
(**a**) Exploratory principal component analysis of metabolite profiles across study groups and (**b**) partial least squares discriminant analysis score plot demonstrating group separation based on metabolite profiles. The blue dots represent the control group and the orange dots represent the GDM group.

A total of 133 distinct metabolites were identified across all the samples. Principal component analysis (PCA) demonstrated moderate clustering between the GDM and control groups. The first two principal components (PC1 and PC2) accounted for 20.63% and 14.0% of the total variance, respectively. Although some overlap was observed, a tendency toward group separation was evident (Figure 1a).

Partial least squares discriminant analysis (PLS–DA) revealed a clearer separation between groups, indicating that the global metabolomic profiles differed between the GDM and control participants. The first two PLS components explained 14% and 16% of the variance, respectively (Figure 1b). Ellipses representing 95% confidence intervals further highlighted distinct group clustering.

### 3.3. Differential Metabolites and Biomarker Identification

Out of 133 metabolites, 10 metabolites showed statistically significant differences (*p* < 0.05) between the GDM and control groups (Table 2). Comprehensive metabolomic data are provided in Table A1.

GC–MS, gas chromatography–mass spectrometry; GDM, gestational diabetes mellitus; MWU, Mann–Whitney U test; TCA, tricarboxylic acid cycle.

**Figure 2:**
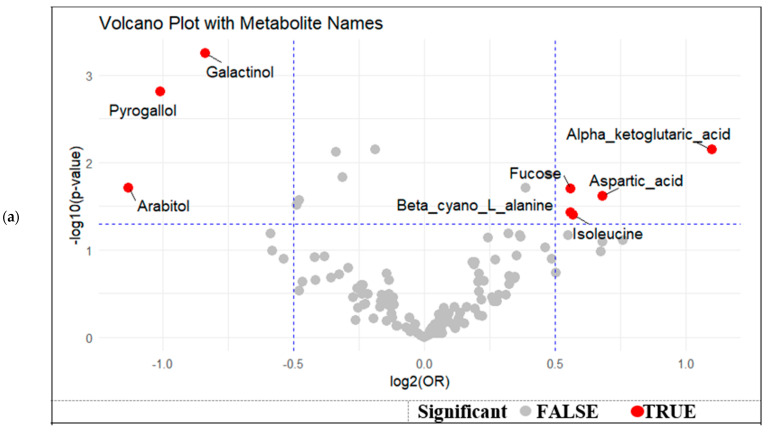

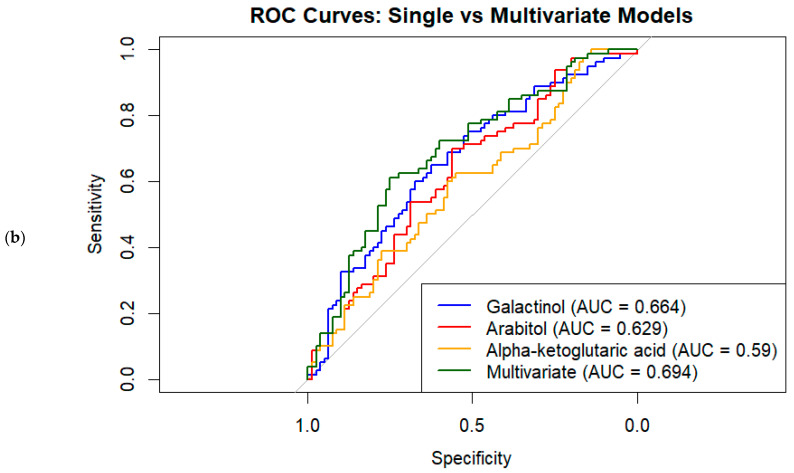
(**a**) Volcano plot of differential metabolites associated with GDM status by effect size and statistical significance, (**b**).

A volcano plot was generated to visualize the effect size (log2 odds ratio) and statistical significance (−log10 *p*-value) of metabolite associations with GDM, adjusted for maternal age, gestational duration, and BMI. Galactinol, pyrogallol, and arabitol were located on the left side of the plot, indicating lower levels in GDM cases (log2[OR] < 0), while maintaining strong statistical significance (Figure 2a). Conversely, metabolites such as Alpha-ketoglutaric acid, aspartic acid, Fucose, and isoleucine were positioned on the right side, indicating a positive association with GDM (log2[OR] > 0). This visual representation underscores a distinct pattern of metabolic dysregulation, with both up- and down-regulated metabolites contributing to the metabolic signature of GDM in breast milk.

Metabolite contributions to group separation were evaluated via VIP (variable importance in projection) scores, where values above 1 (*n* = 44) indicate strong influence on the model. PLS–DA revealed clear separation between GDM and control groups based on metabolomic signatures. Forty-four metabolites showed strong discriminatory power with VIP scores > 1 (Table 3). Notably, galactinol (VIP = 2.50), arabitol (VIP = 2.42), α-ketoglutaric acid (VIP = 2.26), *p*-cresol (VIP = 2.15), and pyrogallol (VIP = 2.14) are the most important contributors to the separation, indicating potential relevance in the metabolic pathways altered in GDM.

**Figure 3:**
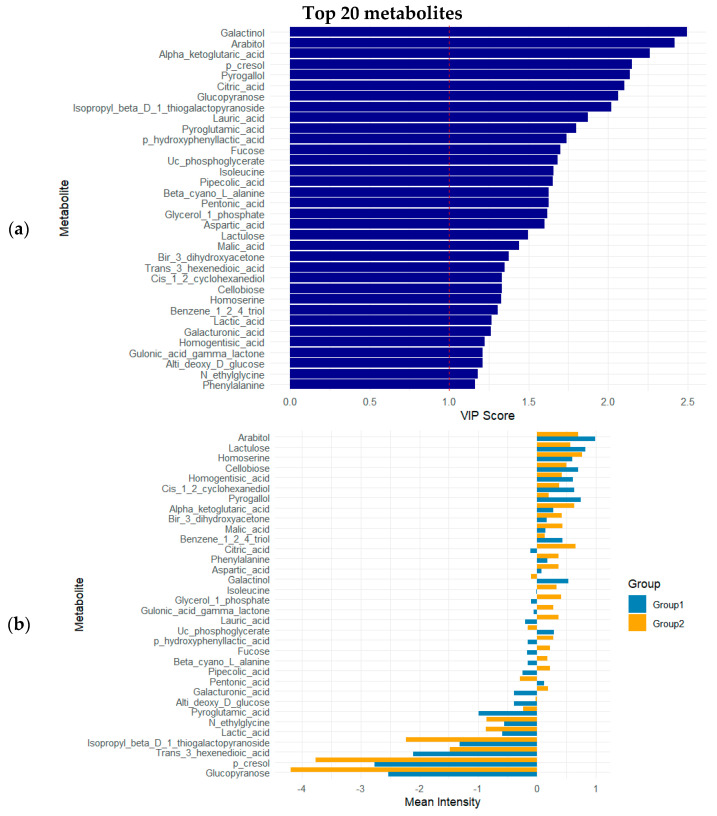
(**a**) VIP scores of top 10 metabolites; (**b**) mean intensities of top 20 VIP metabolites across experimental groups. Group 1: control; Group 2: GDM.

The top 20 variable importance in projection (VIP) metabolites were identified based on their contribution to group separation in the multivariate analysis (Figure 3a). Notably, metabolites such as glucopyranose, trans-3-hexenoic acid, isopropyl beta-D-1-thiogalactopyranoside, and *p*-cresol showed markedly higher mean intensities in Group 2 compared to Group 1 (Figure 3b). Conversely, compounds like arabinitol, lactulose, homoserine, and homogentisic acid demonstrated relatively higher intensities in Group 1.

### 3.4. Predictive Modeling and ROC Analysis

A combination of univariate logistic regression, variable importance in projection (VIP) scores from PLS–DA, and ROC curve analysis identified several metabolites that significantly differed between the GDM and control groups. Among the top metabolites, galactinol (OR = 0.64; 95% CI: 0.49–0.85; *p* = 0.002; VIP = 2.50; AUC = 0.664) and arabitol (OR = 0.37; 95% CI: 0.19–0.72; *p* = 0.003; VIP = 2.42; AUC = 0.629) were notably lower in the GDM group, suggesting potential protective associations. In contrast, Alpha-ketoglutaric acid (OR = 2.00; 95% CI: 1.23–3.28; *p* = 0.006; VIP = 2.26; AUC = 0.590) showed a higher likelihood of being associated with GDM (Table 3).

Univariate ROC analyses of individual metabolites with VIP > 2 showed moderate discriminative ability, with AUC values ranging from 0.566 to 0.664; galactinol achieved the highest AUC of 0.664 (Figure 2b, Table 3). Other significant metabolites with VIP scores > 2.0 included *p*-cresol, pyrogallol, citric acid, and glucopyranose, reflecting their high contribution to group separation in the multivariate model. Notably, several metabolites such as lauric acid, pyroglutamic acid, and isoleucine also demonstrated moderate discriminative ability (AUCs ranging from 0.54 to 0.60), despite their FDR-adjusted *p*-values being slightly above 0.05. The multivariate logistic regression model combining the top five metabolites improved classification performance, yielding an AUC of 0.683, and top three yielding an AUC of 0.694 (Figure 2b).

The Random Forest classifier trained on the same top five metabolites produced a lower AUC of 0.568 on the full dataset. This performance is consistent with the single-run model’s ROC analysis. However, when evaluated using 5-fold cross-validation, the Random Forest model’s average AUC increased to 0.614, indicating moderate predictive performance with some variability across folds. From the initial set of 44 metabolites (VIP > 1), LASSO logistic regression selected a sparse subset as key predictors by shrinking irrelevant coefficients to zero. The logistic regression model built on these selected metabolites achieved a cross-validated average AUC of 0.576, demonstrating modest discriminative ability. The Random Forest model applied on the same dataset and cross-validation procedure showed a slightly higher average AUC of 0.615.

### 3.5. Covariate-Adjusted and FDR-Corrected Analyses

After adjusting for maternal age, gestational duration, and BMI at the time of breast milk sampling, several metabolites remained significantly associated with GDM status at the nominal level (*p* < 0.05; Table 4). However, when applying FDR correction for multiple testing, only galactinol (OR = 0.56; 95% CI: 0.40–0.78; *p* = 0.001; FDR = 0.075) and Pyrogallol (OR = 0.50; 95% CI: 0.32–0.77; *p* = 0.002; FDR = 0.104) retained borderline significance. All other metabolites lost statistical significance after FDR adjustment (FDR > 0.20). This indicates that, although several metabolites show nominal associations, only a small subset remain robust after correction for multiple comparisons, which should be acknowledged as a limitation.

### 3.6. Confusion Matrix and Statistics

The logistic regression model achieved an overall accuracy of 61.25%, correctly classifying approximately 61% of samples (0.5324, 0.6884). Sensitivity was 75.0%, indicating the model successfully identified 75% of GDM cases. Specificity was 47.5%, reflecting lower accuracy in identifying control subjects. The positive predictive value was 58.8%, and the negative predictive value was 65.5%, demonstrating moderate precision for positive and negative classifications. The balanced accuracy was 61.25%, accounting for class imbalance. The Cohen’s Kappa coefficient was 0.225, indicating fair agreement beyond chance between predicted and actual classifications. McNemar’s test revealed a significant difference in misclassification errors between classes (*p* = 0.0077). These results indicate moderate discriminative ability of the logistic regression model, with higher sensitivity than specificity.

### 3.7. Insulin Therapy and Breast Milk Metabolites in GDM

A series of Mann–Whitney U tests were conducted to evaluate the differences in breast milk metabolite levels between GDM mothers with and without insulin use (Table 5). Table A2 provides a comprehensive overview of all the metabolites analyzed in relation to insulin therapy status. Significant differences were observed in several key metabolites involved in amino acid metabolism, glycolysis, the TCA cycle, and oxidative pathways. Allothreonine levels were significantly lower in the insulin-treated group (*p* = 0.005). Xanthine, a purine metabolism intermediate, was significantly higher in the insulin group (*p* = 0.011), indicating a potential increase in oxidative stress markers. Glycerone phosphoric acid, a glycolytic intermediate, also differed significantly (*p* = 0.025), with higher levels in the insulin-treated group. Gluconic acid lactone, a product of glucose oxidation, was elevated in insulin users (*p* = 0.027). Significant increases were also observed for trehalose (*p* = 0.030) and allose (*p* = 0.036), both rare sugars linked to cellular energy regulation and stress responses. Paraoxon, an organic acid possibly involved in detoxification pathways, showed significant variation (*p* = 0.036). Finally, isocitric acid, a TCA cycle intermediate, was significantly higher in the insulin-treated group (*p* = 0.040), reflecting potential alterations in mitochondrial energy metabolism.

## 4. Discussion

We found that GDM is associated with distinct alterations in the metabolomic composition of transitional breast milk. We also provide the first data (to our knowledge) on how insulin treatment in GDM mothers may further modulate the milk metabolome. Overall, our results support that maternal metabolic status influences milk biochemistry, though the effect sizes were moderate and many individual metabolite differences did not reach significance after correction for multiple testing. We identified several specific metabolites that differed between GDM and control milk, including compounds related to carbohydrate metabolism, microbial metabolism, and oxidative pathways, and we demonstrated modest predictive performance of a metabolite panel for classifying GDM status. Additionally, within the GDM group, insulin therapy was associated with variations in amino acid, sugar, and TCA cycle metabolites in milk. In interpreting these findings, we have been careful to avoid overstatement: the associations reported are statistical and do not prove causation, and any potential impacts on infants are speculative at this stage.

### 4.1. Alterations in Carbohydrate and Polyol Metabolites

One of the clearest differences observed was the reduction of galactinol in GDM milk. Galactinol is a sugar alcohol involved in the biosynthesis of raffinose-family oligosaccharides, which serve as prebiotics in human milk [24]. Lower galactinol in GDM mothers could reflect perturbations in hexose metabolism under hyperglycemic conditions, and it might also indicate reduced flux into oligosaccharide production pathways. Since certain oligosaccharides and sugar alcohols in milk can shape the infant gut microbiome and immune development, a deficit could conceivably impact the infant. Similarly, arabitol (a microbial-fermentative polyol) was decreased in GDM milk. Arabitol in milk likely originates from maternal diet or gut microbial activity; its lower level may suggest that GDM alters maternal–microbiome interactions, resulting in less microbial production or transfer to milk [25].

These carbohydrate-related changes align with prior findings that differences in some milk sugars and sugar alcohols are associated with infant adiposity later in infancy [26]. Although we did not follow infant outcomes in our study, one can speculate that an infant receiving milk with lower galactinol and arabitol might experience subtle differences in gut microbiota colonization or metabolic signaling that could affect weight gain. This possibility warrants further study.

### 4.2. Microbial and Phenolic Metabolites

We observed significantly lower *p*-cresol and pyrogallol in GDM milk. Both are products of gut microbial metabolism: *p*-cresol derives from bacterial fermentation of tyrosine, and pyrogallol from microbial breakdown of polyphenols such as gallic acid [27]. Their reduced levels in GDM milk suggest an altered maternal gut microbiome or metabolic output in the context of GDM. Notably, *p*-cresol is usually present in circulation as its sulfate conjugate and has been implicated in immune modulation in infants. A lower level of *p*-cresol in milk may indicate that infants of GDM mothers receive less of this microbial byproduct, which might influence neonatal immune development or microbial colonization of the gut.

Pyrogallol has dual redox properties—it can generate reactive oxygen species but also activate antioxidant pathways (like Nrf2) at low doses. Indeed, one study showed that pyrogallol is a potent inducer of Nrf2-driven antioxidant gene expression [28]. The approximately 40% reduction of pyrogallol in GDM milk could thus reflect a state of oxidative stress in the diabetic mothers (i.e., lower excretion of antioxidant metabolites) and might deprive the infant of certain beneficial redox-active compounds. These findings echo those of Bardanzellu et al., who noted that GDM alters some human milk metabolites related to oxidative stress and inflammation [12]. Taken together, the diminished presence of gut microbiota-derived metabolites (like *p*-cresol and pyrogallol) in GDM milk suggests that maternal dysbiosis or metabolic changes in GDM reduce the transfer of these compounds into milk, which could potentially influence the infant’s oxidative stress balance or gut environment.

### 4.3. Energy Metabolites (TCA Cycle and Lipids)

In contrast to the above changes, we found higher levels of certain TCA cycle intermediates in GDM milk—most prominently α-KG and citric acid, with a trend toward higher isocitric acid. α-KG sits at the crossroads of the TCA cycle and amino acid metabolism; elevated milk α-KG may indicate that diabetic mothers have increased mitochondrial or anaplerotic activity, perhaps due to hyperglycemia driving more substrate through the TCA cycle. Interestingly, a metabolomics study of maternal blood by Pinto et al. noted lower α-KG early in pregnancies that later developed GDM, and another study found altered α-KG levels in cord blood from diabetic pregnancies [18,29]. Our finding of high α-KG in milk at ~2 weeks postpartum suggests that by the lactation stage, mothers with GDM (especially if poorly controlled) might experience a rebound or overshoot in TCA cycle activity. α-KG is also a co-factor for certain epigenetic enzymes (α-KG-dependent dioxygenases) that regulate DNA methylation; thus, variations in milk α-KG might conceivably influence infant epigenetics if α-KG in milk survives digestion and is absorbed, though this remains speculative [30].

Elevated citric acid in GDM milk similarly points to altered energy metabolism. Citrate accumulation could indicate excess citrate being exported from mitochondria (since citrate can inhibit glycolysis and promote lipogenesis). Indeed, GDM milk’s higher citrate might reflect a shift toward lipid synthesis pathways, consistent with some studies reporting higher fat content or lipid metabolites in milk from diabetic mothers. This is in line with Yao et al., who observed increased fatty acid-related metabolites in mature milk of GDM mothers [21]. While we did not directly measure milk fat content, the metabolic profile we observed (high citrate, high α-KG) suggests a reprogramming of mammary energy metabolism in GDM.

### 4.4. Amino Acid and Other Changes

We noted a modest increase in aspartic acid and a decrease in a thiogalactoside (isopropyl-β-D-thiogalactopyranoside) in GDM milk, though the significance of these changes is unclear. Aspartate is a gluconeogenic amino acid; a slight elevation in GDM milk could tie into the altered TCA cycle activity (as aspartate can be derived from oxaloacetate in the TCA cycle). The thiogalactoside is likely a xenobiotic or dietary component (perhaps derived from certain foods or gut bacterial metabolism); its lower level in GDM milk could reflect differences in maternal diet or gut microbial processing between groups. Additionally, we observed higher levels of α-santonin (a plant-derived compound, possibly from maternal diet or herbal remedies) in GDM milk. We hesitate to overinterpret the α-santonin finding, but its presence indicates how maternal diet or supplement habits (e.g., consumption of certain herbs or teas) could differ between the GDM and control groups. GDM mothers might have taken specific herbal remedies or had unmeasured dietary differences—an example of a potential confounder in metabolomic studies. Unfortunately, we lacked detailed maternal dietary intake data to explore this further, which is a limitation of our study.

### 4.5. Comparison with Previous Studies

Our results corroborate and extend earlier studies of milk metabolomics in GDM. For example, Wen et al. reported that the milk of GDM mothers showed distinct metabolite trajectories over lactation, including more pronounced changes in lipid and amino acid metabolites compared to non-GDM mothers [20]. In particular, our data focusing on the transitional milk phase (~2 weeks postpartum) align with the notion that this period is when GDM-related differences in milk composition are most evident. Indeed, Wu et al. found the largest number of differing metabolites in transitional milk (with 30 compounds lower and 19 higher in GDM), whereas fewer differences were observed in colostrum or mature milk [19]. We likewise observed multiple composition differences at 10–15 days postpartum, affirming transitional milk as a sensitive window for detecting the impact of GDM on milk biochemistry.

We also identified similar classes of metabolites affected by GDM as reported in previous studies. For instance, Wu et al. noted reductions in certain glycerophospholipids and fatty acids in GDM milk, whereas we found reductions in *p*-cresol and galactinol (which, while not lipids, relate to gut and carbohydrate metabolism) and increases in TCA cycle intermediates (suggesting altered energy utilization). Our multivariate results mirror those of Wu et al., in that a combination of milk metabolites can distinguish GDM status with only moderate accuracy. Their metabolite panel achieved an ROC AUC of around 0.75, slightly higher than our panel’s AUC of ~0.68, but both indicate only partial separation between GDM and control milk profiles [19]. Moreover, Wu et al. observed correlations between concentrations of certain milk metabolites (e.g., long-chain fatty acids, lysophospholipids) and infant growth outcomes [19]. We did not have data on infant growth or body composition, but the fact that many GDM-associated milk metabolites in our study are involved in energy and redox balance suggests plausible links to infant metabolic health.

Notably, Nagel et al. recently demonstrated that the metabolomic profile of milk from GDM mothers is associated with greater infant adiposity in early life [26]. In that U.S. cohort, differences in milk nucleotide derivatives, one-carbon metabolites, and fatty acids distinguished GDM milk, and infants who consumed milk from GDM mothers had higher fat mass indices by 4–8 weeks of age [26]. This finding implicates milk composition as one potential mediator between maternal GDM and higher obesity risk in the offspring. Our study reinforces this concept by identifying which specific metabolites are altered in GDM milk; it also raises a new consideration that prior studies did not address—namely, that insulin treatment in GDM mothers could further modify these exposures.

### 4.6. Impact of Insulin Therapy

A novel aspect of our work is the analysis of milk from insulin-treated versus diet-controlled GDM mothers. Interestingly, we found that insulin therapy was associated with further shifts in the milk metabolome—notably, in our GDM subgroup, the insulin-treated mothers’ milk showed lower levels of certain metabolites (e.g., allothreonine) and higher levels of others (e.g., xanthine, trehalose, allose, glycerone phosphate, isocitrate) compared to the milk of GDM mothers managed with diet alone. These changes suggest that pharmacologic insulin, while improving glycemic control, might induce unique metabolic states in the lactating mother. Insulin has anabolic effects that could increase the uptake of certain amino acids from plasma into maternal tissues (potentially explaining the lower allothreonine observed in milk). It also influences glucose utilization and could enhance certain alternative pathways in the mammary gland—for example, the elevated glycerone-P and trehalose in insulin-treated GDM milk might reflect increased glucose flux being diverted into auxiliary sugar metabolism pathways under insulin stimulation. Some of the changes we observed (such as higher xanthine and isocitrate) might be markers of more severe hyperglycemia or diabetes in the insulin-requiring mothers, rather than effects of insulin per se. For instance, a high level of xanthine is suggestive of oxidative stress; the insulin-treated mothers in our study may have had poorer glycemic control prior to insulin initiation, leading to greater oxidative stress despite eventual insulin use [31]. Without direct measures of oxidative stress or glycemic excursions, we cannot confirm this, but it is a plausible interpretation.

The key point is that insulin treatment for GDM is not metabolically “neutral”—it appears to alter the profile of milk metabolites, which could have implications for the nursing infant. On one hand, better glycemic control from insulin might normalize some aspects of milk composition; on the other hand, exogenous insulin might introduce changes such as altered amino acid or nucleotide levels that would not occur with diet therapy alone. The net impact of these differences on the infant is unknown. Hypothetically, the lower essential amino acid (allothreonine) in insulin-treated GDM milk could affect infant protein nutrition, and the higher xanthine could expose the infant to more pro-oxidant or pro-oxidative signals, but these possibilities are speculative. Our data on insulin-treated vs. non-insulin GDM are preliminary and require replication in larger cohorts, but they highlight the need to consider maternal treatment modality as a factor in human milk studies. To our knowledge, previous milk metabolomics studies in GDM have not examined this aspect—for example, the reports by Wu, Wen, and Yao did not include stratified analyses by insulin use [19,20,21]. Our findings encourage future research to specifically compare diet-controlled versus insulin-requiring GDM in terms of milk composition and to evaluate whether any such differences translate into differences in infant outcomes.

### 4.7. Potential Implications for Infants

The ultimate concern is whether these metabolomic differences in milk have any impact on infants born to GDM mothers. Infants of diabetic pregnancies are known to be at higher risk for rapid postnatal weight gain and childhood obesity [2]. Breastfeeding has well-documented benefits for infant health, and it is conceivable that milk from GDM mothers might carry subtle “signals” that influence the infant’s metabolism.

For instance, reduced levels of prebiotic sugars and microbial metabolites in GDM milk could alter the infant’s gut microbiome colonization, possibly affecting energy harvest or inflammatory tone in the infant. Likewise, changes in energy-related metabolites (such as the higher citrate and α-KG, or differences in amino acid-derived metabolites) might affect the infant’s metabolic hormone regulation or nutrient sensing. Some milk metabolites can act as signaling molecules; for example, citrate can chelate minerals and modulate fatty acid synthesis, and α-KG can influence cellular signaling and epigenetic enzymes in tissues. Our data also hint that infants of insulin-treated GDM mothers receive milk with a somewhat different metabolic makeup than those of diet-treated GDM mothers—adding another layer of complexity to consider in early nutrition for these infants.

Nagel et al. provided empirical evidence for such infant impacts: they found that the metabolomic profile of milk from GDM mothers was associated with greater adiposity in their infants by 1–2 months of age [26]. In that study, infants who consumed milk from GDM mothers had higher fat mass indices, supporting the idea that milk composition may mediate some of the relationship between maternal GDM and infant obesity risk [26]. Our study strengthens this concept by detailing the specific metabolite alterations in GDM milk (and by suggesting that maternal insulin treatment may further influence these metabolite patterns). However, it is important to stress that at this point any implications for infants are hypotheses only.

The observational nature of our study precludes concluding any causal effect on infants. Many factors influence infant growth and development (including genetics, overall nutrition, and environment), and breast milk composition is just one piece of that puzzle. Thus, while we have discussed potential mechanisms (microbiome modulation, metabolic signaling, etc.), these should not be overstated. It is equally possible that infants adapt to these milk differences or that the magnitude of the differences is too small to cause measurable effects.

Crucially, our results do not imply that breast milk from GDM mothers is “harmful” or should be avoided—on the contrary, breastfeeding remains strongly recommended for women with GDM, as it can reduce the infant’s risk of obesity and diabetes relative to formula feeding. If anything, our findings might eventually guide targeted enhancements or interventions. For example, Wu et al. speculated about supplementing or fortifying the milk of diabetic mothers with certain beneficial sugars that tend to be lower in GDM milk [19]. We observed similar deficits (e.g., in galactinol and other milk sugars), but at this point such interventions are purely theoretical. Any suggestion to alter human milk composition should be approached with extreme caution and only after thorough evidence of benefit, given that breast milk is a highly evolved, complex fluid optimized by nature for infant nutrition.

### 4.8. Limitations

This study has several limitations. Its observational design precludes any conclusions about causality—associations between GDM and milk metabolites could be influenced by unmeasured factors such as maternal diet, genetics, or microbiome differences. We did not collect detailed dietary intake data or gut microbiota profiles, which could have provided insight into sources of certain metabolites. We also lacked measurements of maternal metabolic status at the time of milk collection (e.g., blood glucose or insulin levels), so we cannot correlate milk changes with the degree of glycemic control. The reliance on self-reported medication and supplement use is a limitation; although we attempted to verify and exclude recent medication use, we cannot rule out recall bias or undisclosed use of substances that might affect milk composition. Our sample collection was cross-sectional at one lactation stage; a longitudinal design would better capture dynamic changes and confirm whether differences persist or diminish in later mature milk. The sample size, while sufficient for primary comparisons, was relatively small for subgroup analysis and for detecting smaller effect sizes—some true differences may have gone undetected due to limited power. Additionally, the multivariate model performance indicates risk of overfitting, and our results (especially the predictive biomarkers) should be validated in independent cohorts. On the analytical side, our GC–MS approach covered primarily polar metabolites and required derivatization; some compounds may not have been detected or accurately quantified (e.g., certain sugars can be tricky to resolve). We mitigated technical variability with rigorous QC, but subtle batch effects are always possible. Lastly, while we speculate on potential impacts on infants, we did not measure infant outcomes in this study. Thus, any discussion of infant health implications is hypothetical and based on the literature, not on direct evidence from our cohort.

## 5. Conclusions

In conclusion, this study identifies a distinctive metabolomic fingerprint in transitional breast milk from mothers with gestational diabetes. Key alterations were noted in pathways of carbohydrate (glycoside) metabolism, microbial metabolites linked to oxidative stress, and mitochondrial energy substrates. These disruptions suggest that maternal GDM status is reflected in the milk’s biochemical composition in ways that could be biologically meaningful. We also found that insulin therapy for GDM is associated with further metabolic shifts in milk, highlighting that treatment strategies might have downstream effects on milk composition. Taken together, our findings underscore the importance of considering maternal metabolic health in lactation research. They provide a foundation for future work to determine how these milk compositional changes might affect infant outcomes and whether any interventions (maternal dietary guidance, milk fortification, etc.) could help optimize breast milk composition for infants’ health. Ultimately, unraveling the connections between maternal metabolism, milk constituents, and infant development could lead to targeted approaches to improve the health of children born to mothers with GDM.

## Figures and Tables

**Table 1:** Mother/newborn characteristics in the study groups.

	Control (*n* = 80)	GDM (*n* = 80)	*p*
Maternal age (years)	29.2 ± 5.4	31.3 ± 5.3	0.018
Education, high school or above (%)	60.0	63.7	0.625
Working status	16.3	22.5	0.317
Monthly income			0.395
Below minimum wage	12.5	12.5	
Minimum wage–2× minimum wage	50.0	37.5	
2× minimum wage–3× minimum wage	31.3	40.0	
3× minimum wage and above	6.3	10.0	
Smoking (%)	22.5	20.3	0.730
Passive smoking (%)	32.5	41.3	0.251
Insulin therapy		61.3	
Supplementation during pregnancy			
Folic acid supplementation	83.8	77.5	0.317
Multivitamin supplementation	53.8	43.8	0.206
Vitamin D supplementation	48.8	58.8	0.205
Preconceptional BMI (kg/m^2^)	25.7 ± 5.3	30.1 ± 5.9	<0.001
Postnatal BMI (kg/m^2^), PP10–15d	28.0 ± 5.4	31.1 ± 5.6	0.001
Weight gain according to preconceptional BMI			0.729
Insufficient	22.5	18.8	
Adequate	26.3	31.3	
Excessive	51.2	50.0	
Polyhydramnios history in this pregnancy	3.8	21.3	0.001
Mode of delivery, Cesarean section (%)	82.4	81.2	0.271
Infant characteristics			
Birth order, the first	21.3	22.5	0.848
Male sex (%)	45.0	52.5	0.343
Gestational duration, week	38.5 ± 1.2	37.7 ± 1.3	<0.001
Birth weight according to gestational duration			0.005
SGA	5.1	2.5	
AGA	89.8	75.0	
LGA	5.1	22.5	
Birth weight Z-score	0.14 ± 0.77	0.51 ± 1.02	0.010

Values are presented as mean ± SD or (%).

**Table 2:** Metabolites identified by GC–MS analysis and differences in their relative concentrations according to groups.

Metabolite	Biochemical Class	Control	GDM	MWU. *p*
Aspartic acid	Amino acids	0.17 [1.2]	0.29 [1.94]	0.045
D-Glucopyranose	Monosaccharide/glucose derivative	0 [2.59]	0 [0]	0.046
α-Santonin	Sesquiterpene lactone	0.35 [0.83]	0.68 [1.34]	0.042
*p*-Cresol	Phenolic compound (para-cresol, a monocyclic phenol)	0.01 [0.05]	0 [0.02]	0.019
*p*-Hydroxyphenyllactic acid	Aromatic organic acid	0.3 [0.85]	0.49 [1.87]	0.037
Pyrogallol	Polyphenol	0.68 [1.26]	0.39 [0.76]	0.014
trans-3-Hexenedioic acid	Unsaturated dicarboxylic acid (medium-chain fatty acid derivative)	0.01 [0.03]	0.02 [0.25]	0.039
Galactinol	Glycoside	0.74 [1.55]	0.37 [0.77]	0.003
Isopropyl β-D-1-thiogalactopyranoside	Thioglycoside	0.1 [0.45]	0.04 [0.16]	0.031
Citric acid	Tricarboxylic acid (TCA cycle intermediate)	0.56 [0.77]	0.79 [0.77]	0.009

Values are presented as the median [IQR].

**Table 3:** Top differential metabolites between GDM and control groups with associated statistics: odds ratios, VIP scores, and predictive performance.

Metabolite	OR	95% CI	*p*	FDR	VIP	AUC
Galactinol	0.64	0.49–0.85	0.002	0.167	2.50	0.664
Arabitol	0.37	0.19–0.72	0.003	0.167	2.42	0.629
α-Ketoglutaric acid	2.00	1.23–3.28	0.006	0.167	2.26	0.590
*p*-Cresol	0.81	0.69–0.94	0.008	0.167	2.15	0.631
Pyrogallol	0.59	0.4–0.85	0.005	0.167	2.14	0.646
Citric acid	1.31	1.07–1.6	0.009	0.167	2.11	0.615
D-Glucopyranose	0.89	0.82–0.97	0.008	0.167	2.06	0.613
Isopropyl β-D-1-thiogalactopyranoside	0.82	0.71–0.95	0.010	0.168	2.02	0.605
Lauric acid	1.33	1.05–1.69	0.019	0.277	1.87	0.600
Pyroglutamic acid (5-Oxoproline)	1.21	1.03–1.42	0.022	0.277	1.80	0.566
*p*-Hydroxyphenyllactic acid	1.36	1.04–1.78	0.026	0.277	1.74	0.591
L-Fucose	1.38	1.03–1.84	0.029	0.277	1.70	0.596
3-Phosphoglycerate	0.75	0.58–0.97	0.031	0.277	1.68	0.598
L-Isoleucine	1.43	1.03–1.99	0.034	0.277	1.66	0.590
Pipecolic acid	1.29	1.02–1.64	0.035	0.277	1.65	0.544
β-Cyano-L-alanine	1.41	1.02–1.96	0.036	0.277	1.63	0.598
Pentonic acid	0.76	0.58–0.98	0.037	0.277	1.63	0.568
Glycerol 1-phosphate	1.25	1.01–1.55	0.038	0.277	1.62	0.580
L-Aspartic acid	1.46	1.02–2.11	0.039	0.277	1.60	0.589

Odds ratios (ORs) < 1 indicate lower metabolite levels in GDM vs. control; ORs > 1 indicate higher in GDM; 95% CI: confidence interval for OR. *p*-values from logistic regression of GDM status on each metabolite (unadjusted), with FDR-adjusted q-values. VIP: variable importance in projection score from PLS–DA. AUC: Area Under ROC Curve if that metabolite is used alone as a classifier. For reference, a multivariate model combining the top 5 metabolites had AUC ≈ 0.68 (full data) and ~0.58 after cross-validation, see text.

**Table 4:** Multivariate logistic regression for associations between breast milk key metabolites and GDM status controlling for maternal age, gestational duration, and BMI.

Metabolite	OR	95% CI	*p*	FDR
α-Ketoglutaric acid	2.14	1.23–3.73	0.007	0.202
Arabitol	0.46	0.24–0.88	0.019	0.269
L-Aspartic acid	1.60	1.06–2.41	0.024	0.295
β-Cyano-L-alanine	1.47	1.02–2.12	0.037	0.356
Citric acid	1.31	1.04–1.64	0.019	0.269
L-Fucose	1.47	1.06–2.04	0.020	0.269
Galactinol	0.56	0.4–0.78	0.001	0.075
Glucoheptonic acid	0.71	0.52–0.97	0.030	0.314
D-Glucopyranose	0.88	0.8–0.97	0.007	0.202
L-Isoleucine	1.48	1.02–2.16	0.040	0.356
*p*- Isopropyl β-D-1-thiogalactopyranoside	0.79	0.67–0.94	0.008	0.202
Lauric acid	1.39	1.07–1.8	0.014	0.269
*p*-Cresol	0.80	0.68–0.96	0.015	0.269
Pentonic acid	0.72	0.54–0.96	0.027	0.303
Pyrogallol	0.50	0.32–0.77	0.002	0.104

GDM, gestational diabetes mellitus; BMI, Body mass index; OR, odds ratio; 95% CI, 95% confidence interval; *p*, *p*-value; FDR, False Discovery Rate.

**Table 5:** Significant metabolic profile differences according to insulin therapy in GDM cases.

	Absence	Presence	MWU, *p*
	*n* = 31	*n* = 49	
Allothreonine	1.15 [1.00]	0.77 [0.61]	0.005
Allose	0.45 [0.57]	0.69 [1.02]	0.036
Glycerone phosphoric acid	0.02 [0.04]	0.06 [0.17]	0.025
Trehalose	0.16 [0.29]	0.28 [0.49]	0.030
Gluconic acid lactone	0.17 [0.77]	0.61 [1.27]	0.027
Paraoxon	0.89 [0.99]	1.12 [0.54]	0.036
Xanthine	0.10 [0.37]	0.24 [0.92]	0.011
Isocitric acid	0.13 [0.39]	0.32 [0.52]	0.040

GDM, gestational diabetes mellitus; MWU, Mann–Whitney U test.

## Data Availability

The raw data supporting the conclusions of this article will be made available by the corresponding authors on request.

## Appendix A

**Table A1:** Metabolites identified by GC–MS analysis and differences in their relative concentrations according to groups.

Metabolites	Biochemical Class	Control	GDM	MWU. *p*
Acetanilide	Aniline derivative	0.25 [0.70]	0.41 [0.95]	0.282
α-D-Glucosamine 1-phosphate	Amino sugar phosphate	0.28 [1.91]	0.34 [1.29]	0.638
Asp-Glu (aspartyl-glutamate)	Dipeptide	0.46 [0.95]	0.66 [1.49]	0.152
β-Cyano-L-alanine	L-α-Amino acids	0.14 [0.85]	0.32 [1.46]	0.101
Cysteinylglycine	Dipeptide	0.05 [0.07]	0.05 [0.07]	0.718
2-Amino-1-phenylethanol	Aralkylamines	0.32 [2.23]	0.42 [2.14]	0.867
2-Piperidone	Cyclic amide	0.39 [0.89]	0.33 [1.61]	0.415
Lyxosylamine	Glycosylamine	0.34 [1.36]	0.3 [1.14]	0.787
*N*-Ethylglycine	α-Amino acids	0.08 [0.12]	0.08 [0.07]	0.806
Pyroglutamic acid (5-oxoproline)	Proline and derivatives	0.22 [1.23]	0.34 [1.23]	0.187
3-Methyl-2-oxobutanoic acid	Short-chain keto acids	0.51 [0.89]	0.56 [0.75]	0.843
Alanine	Alanine and derivatives	0.09 [0.32]	0.14 [0.75]	0.204
Allothreonine	L-α-Amino acids	0.93 [0.91]	0.96 [0.92]	0.743
Aspartic acid	Aspartic acid and derivatives	0.17 [1.2]	0.29 [1.94]	0.045
5-Aminovaleric acid	δ-Amino acids	0.28 [0.79]	0.51 [0.92]	0.134
Citrulline	L-α-Amino acids	0.29 [1.26]	0.56 [1.66]	0.768
Glutaric acid (not listed, but pimelic below)	Dicarboxylic acid	0.23 [0.94]	0.37 [1.15]	0.180
Glycine	α-Amino acids	0.08 [0.16]	0.08 [0.31]	0.322
Homoserine	L-α-Amino acids	0.49 [1.36]	0.57 [1.37]	0.296
Isoleucine	Isoleucine and derivatives	0.18 [1.35]	0.42 [1.5]	0.084
Phenylalanine	Phenylalanine and derivatives	0.16 [1.23]	0.25 [1.54]	0.144
Proline	Proline and derivatives	0.39 [0.77]	0.49 [0.84]	0.161
Serine	Serine and derivatives	0.48 [1.78]	0.46 [1.41]	0.984
Threonine	L-α-Amino acids	0.23 [1.57]	0.28 [1.5]	0.311
Tyramine	Phenethylamines	0.33 [0.37]	0.32 [0.44]	0.772
Valine	Valine and derivatives	0.35 [1.52]	0.42 [1.53]	0.380
Allo-Inositol	Cyclitol	0.02 [0.04]	0.04 [0.06]	0.122
Ascorbic acid (Vitamin C)	Organic acid	0.09 [0.51]	0.07 [0.24]	0.854
5-Aminoimidazole-4-carboxamide	Aminoimidazoles	0.21 [0.33]	0.2 [0.25]	0.921
Pantothenic acid	β-Amino acids and derivatives	0.62 [0.8]	0.68 [1.04]	0.616
Arabitol (Arabitol = Arabinol)	Sugar alcohol	0.88 [1.07]	0.53 [1.05]	0.072
Benzene-1,2,4-triol (Hydroxyquinol)	Phenolic compound	0.49 [1.6]	0.38 [1.37]	0.399
(1R,2S)-cis-1,2-dihydro-1,2-naphthalenediol	Naphthalenes	0.34 [1.72]	0.36 [1.84]	0.392
cis-1,2-Cyclohexanediol	Cyclohexanediols	0.49 [1.58]	0.44 [0.98]	0.423
Dithiothreitol (DTT)	Thiol derivative of a sugar alcohol	1 [0.6]	1.01 [0.47]	0.712
Glycerol	Sugar alcohol	0.82 [0.99]	0.92 [1.53]	0.602
2-Butyne-1,4-diol	Diol	0.01 [0.01]	0.01 [0.01]	0.643
Ribitol (Adonitol)	Sugar alcohol	0.34 [0.86]	0.37 [0.86]	0.495
Sorbitol	Sugar alcohol	0.23 [0.92]	0.26 [0.96]	0.427
Threitol	Sugar alcohol	0.53 [0.86]	0.48 [0.8]	0.854
Acetol (Hydroxyacetone)	α-Hydroxy ketone	0.12 [0.41]	0.08 [0.4]	0.824
Allose	Monosaccharide	0.38 [0.8]	0.55 [0.7]	0.053
6-Deoxy-D-glucose	Monosaccharide	0.18 [1.04]	0.36 [1.49]	0.172
1,3-Dihydroxyacetone	Monosaccharide	0.27 [1.61]	0.36 [0.99]	0.266
Fructose	Monosaccharide	0.79 [1.08]	0.71 [0.93]	0.720
Glucopyranose	Monosaccharide	0 [2.59]	0 [0]	0.046
Glyceraldehyde	Monosaccharide	0.82 [1]	0.94 [1.59]	0.606
Glycerol 1-phosphate	Sugar alcohol phosphate	0.34 [0.7]	0.55 [0.72]	0.109
Glycerone phosphate	Ketose phosphate	0.03 [0.08]	0.04 [0.13]	0.859
Lactose	Disaccharide	0.12 [0.63]	0.14 [1.38]	0.970
Mannose	Monosaccharide	0.48 [0.95]	0.57 [1.2]	0.425
Sucrose	Disaccharide	0.3 [1.1]	0.28 [1.4]	0.659
Trehalose	Disaccharide	0.19 [0.48]	0.21 [0.3]	0.417
3-Phosphoglycerate	Sugar acids and derivatives	0.44 [1.47]	0.19 [1.04]	0.201
3-Phosphoglyceric acid	Organic acid phosphate	0.65 [0.66]	0.78 [0.99]	0.162
9-Fluorenone	Polycyclic aromatic ketone	0.37 [1.8]	0.29 [0.67]	0.318
2′-Deoxycytidine	Nucleoside	0.11 [0.12]	0.11 [0.11]	0.986
Thymine	Nucleobase	0.16 [2.25]	0.23 [1.9]	0.822
Alpha-Santonin	Sesquiterpene lactone	0.35 [0.83]	0.68 [1.34]	0.042
Benzoic acid	Aromatic carboxylic acid	0.65 [0.99]	0.75 [1.68]	0.560
1-Aminocyclopropane-1-carboxylic acid	α-Amino acids	0.77 [1.03]	0.53 [0.73]	0.249
Dehydroascorbic acid	Oxidized form of vitamin C	1 [0.8]	0.91 [0.8]	0.467
Galacturonic acid	Uronic acid	0.24 [1]	0.49 [1.45]	0.096
Glucoheptonic acid	Sugar acid	0.25 [1]	0.19 [0.87]	0.730
Gluconic acid	Aldonic acid	0.22 [0.37]	0.28 [0.21]	0.597
Gluconic acid lactone	Lactone form of gluconic acid	0.27 [1.04]	0.29 [1.1]	0.814
Glucosaminic acid	Amino sugar acid	0.39 [1.22]	0.43 [1.38]	0.226
Glucuronic acid	Uronic acid	0.49 [1.79]	0.5 [1.81]	0.553
Glyceric acid	Sugar acid	0.25 [1.97]	0.28 [1.99]	0.510
Glycolic acid	α-Hydroxy acid	0.09 [1.81]	0.08 [1.32]	0.851
Gulonic acid γ-lactone	Lactone of gulonic acid	0.44 [1.17]	0.48 [1.88]	0.322
Homogentisic acid	Aromatic acid	0.56 [1.22]	0.45 [0.71]	0.618
Itaconic acid	Dicarboxylic acid	0.51 [1.59]	0.63 [1.75]	0.204
Lactamide	Amide	0.58 [0.79]	0.48 [0.62]	0.267
Lactic acid	α-Hydroxy acid	0.12 [0.18]	0.1 [0.15]	0.343
Lactobionic acid	Fatty acyl glycosides of saccharides	0.32 [1.62]	0.21 [0.89]	0.623
Lactulose	Disaccharide	0.58 [1.35]	0.49 [0.82]	0.350
Lyxose	Monosaccharide	0.65 [1.53]	0.59 [1.82]	0.695
Maleamic acid	Unsaturated amide	0.32 [1.49]	0.44 [1.74]	0.179
Malonic acid	Dicarboxylic acid	0.28 [2.02]	0.4 [1.78]	0.680
Methyl-β-D-galactopyranoside	O-Glycosyl compounds	0.57 [0.89]	0.48 [1]	0.897
Methylmalonic acid	Dicarboxylic acids and derivatives	0.67 [1.46]	0.45 [1.41]	0.986
Neohesperidin	Flavonoid glycoside	0.03 [0.25]	0.05 [0.57]	0.125
O-Phosphocolamine (O-phosphoethanolamine)	Phosphoethanolamines	0.14 [0.42]	0.2 [1.32]	0.474
10-Hydroxydecanoic acid	Medium-chain hydroxy acids	0.41 [1.48]	0.5 [1.49]	0.875
Oxalic acid	Dicarboxylic acid	0.77 [1.18]	0.82 [1.55]	0.774
*p*-Cresol (4-methylphenol)	Phenolic compound	0.01 [0.05]	0 [0.02]	0.019
*p*-Hydroxyphenyllactic acid	Aromatic α-hydroxy acid	0.3 [0.85]	0.49 [1.87]	0.037
Paraoxon	Organophosphate compound	1.11 [0.96]	1.07 [0.87]	0.705
Pentonic acid	Sugar acids and derivatives	0.25 [0.35]	0.18 [0.3]	0.068
Phenyl-β-D-glucopyranoside	Phenolic glycosides	0.28 [1.78]	0.37 [1.41]	0.793
Phosphoric acid (orthophosphate)	Phosphorus oxoacid	0.29 [0.23]	0.26 [0.16]	0.380
Pi (inorganic phosphate)	Inorganic phosphate	0.01 [0.1]	0.01 [0.12]	0.883
Pimelic acid	Dicarboxylic acid	0.15 [2.11]	0.07 [2.44]	0.550
Pipecolic acid	L-α-Amino acids	0.26 [1.92]	0.33 [1.86]	0.278
Porphine	Porphyrin	0.43 [1.01]	0.43 [1.25]	0.455
Pyrogallol (1,2,3-benzenetriol)	Phenolic compound	0.68 [1.26]	0.39 [0.76]	0.014
Quinic acid	Cyclohexanecarboxylic acid	0.46 [0.9]	0.39 [1.26]	0.986
Ribonic acid γ-lactone	Lactone of ribonic acid	0.64 [1.23]	0.67 [1.27]	0.640
Sedoheptulose anhydride	Sugar derivative	0.12 [0.47]	0.13 [1.44]	0.224
Shikimic acid	Cyclohexene triol carboxylic acid	0.68 [0.76]	0.59 [0.47]	0.482
Sorbose	Monosaccharide	0.19 [0.4]	0.38 [0.65]	0.056
Sphingosine	Long-chain amino alcohol	0.03 [0.15]	0.05 [0.43]	0.117
Succinic acid	Dicarboxylic acid	0.24 [1.75]	0.33 [1.9]	0.397
Tartaric acid	Dihydroxy dicarboxylic acid	0.27 [0.48]	0.26 [0.85]	0.655
Tartronic acid	Hydroxy dicarboxylic acid	0.19 [1.53]	0.31 [1.59]	0.331
Threose	Monosaccharide	0.7 [1.46]	0.8 [1.72]	0.728
Trans-3-hexenedioic acid	Dicarboxylic acid	0.01 [0.03]	0.02 [0.25]	0.039
Urea	Ureas	0.14 [1.31]	0.21 [1.92]	0.382
3-Hydroxypropanoic acid	β-Hydroxy acid	0.86 [1.61]	1.01 [1.72]	0.254
Xanthine	Purine base	0.16 [0.88]	0.22 [0.37]	0.835
Cellobiose	Disaccharide	0.4 [1.48]	0.35 [1.33]	0.495
Fucose	Hexoses	0.17 [1.03]	0.34 [1.42]	0.079
Galactinol	O-Glycosyl compounds	0.74 [1.55]	0.37 [0.77]	0.003
Isopropyl β-D-thiogalactopyranoside (IPTG)	Thiogalactoside	0.1 [0.45]	0.04 [0.16]	0.031
Capric acid (Decanoic acid)	Fatty acid	0.45 [1.33]	0.47 [1.44]	0.560
Caprylic acid (Octanoic acid)	Fatty acid	0.45 [1.87]	0.11 [1.81]	0.555
Lauric acid (Dodecanoic acid)	Fatty acid	0.34 [0.95]	0.47 [0.97]	0.106
Methyl caprylate (Methyl octanoate)	Fatty acid ester	0 [0.02]	0 [0.03]	0.997
Myristic acid (Tetradecanoic)	Fatty acid	0.27 [1.37]	0.4 [1.73]	0.583
Palmitic acid (Hexadecanoic)	Fatty acid	0.49 [1.39]	0.47 [1.63]	0.467
Citramalic acid	Methylmalic acid	0.41 [1.88]	0.53 [1.64]	0.491
α-Ketoglutaric acid	Keto-dicarboxylic acid	0.4 [1.26]	0.49 [1.61]	0.142
Citric acid	Tricarboxylic acid	0.56 [0.77]	0.79 [0.77]	0.009
Erythrose-4-phosphate	Sugar phosphate	1.1 [0.41]	0.99 [0.38]	0.544
Isocitric acid	Tricarboxylic acid	0.22 [0.61]	0.22 [0.48]	0.940
Malic acid	Hydroxy dicarboxylic acid	0.29 [1.25]	0.54 [1.37]	0.198
Oxaloacetic acid	Keto-dicarboxylic acid	0.38 [1.07]	0.35 [0.82]	0.932
Linoleic acid	Fatty acid	0.37 [1.15]	0.38 [1.23]	0.992
Methyl linolenate (Methyl 18:3)	Fatty acid ester	0.49 [0.9]	0.44 [1.17]	0.862
Oleic acid	Fatty acid	0.42 [1.17]	0.36 [1.61]	0.921
Palmitoleic acid	Fatty acid	0.61 [1.12]	0.63 [1.04]	0.878
trans,trans-Farnesol	Sesquiterpene alcohol	0.71 [1.38]	0.62 [1.47]	0.519

The data are presented as the median [IQR]; GC–MS: gas chromatography–mass spectrometry; GDM: gestational diabetes mellitus; MWU: Mann–Whitney U test.

**Table A2:** Comprehensive metabolic profile differences according to insulin therapy in GDM cases.

Metabolites	Biochemical Class	Absence	Presence	MWU, *p*
		*n* = 31	*n* = 49	
Acetanilide	Aniline derivative	0.43 [0.78]	0.38 [1.4]	0.611
α-D-Glucosamine 1-phosphate	Amino sugar phosphate	0.36 [1.04]	0.27 [1.75]	0.756
Asp-Glu (aspartyl-glutamate)	Dipeptide	0.65 [0.89]	0.72 [1.94]	0.824
β-Cyano-L-alanine	L-α-Amino acids	0.31 [1.35]	0.33 [2.08]	0.625
Cysteinylglycine	Dipeptide	0.05 [0.08]	0.04 [0.07]	0.933
2-Amino-1-phenylethanol	Aralkylamines	0.48 [1.69]	0.34 [2.28]	0.604
2-Piperidone	Cyclic amide	0.34 [1.32]	0.33 [1.66]	0.824
Lyxosylamine	Glycosylamine	0.29 [0.81]	0.31 [2.17]	0.816
*N*-Ethylglycine	α-Amino acids	0.1 [0.08]	0.08 [0.07]	0.456
Pyroglutamic acid (5-oxoproline)	Proline and derivatives	0.28 [0.95]	0.51 [1.41]	0.415
3-Methyl-2-oxobutanoic acid	Short-chain keto acids	0.45 [0.78]	0.57 [0.69]	0.611
Alanine	Alanine and derivatives	0.03 [0.53]	0.15 [0.77]	0.201
Allothreonine	L-α-Amino acids	1.15 [1.00]	0.77 [0.61]	0.005
Aspartic acid	Aspartic acid and derivatives	0.25 [1.19]	0.34 [1.95]	0.427
5-Aminovaleric acid	δ-Amino acids	0.47 [1.09]	0.52 [0.85]	0.474
Citrulline	L-α-Amino acids	0.77 [1.49]	0.29 [1.81]	0.855
Glutaric acid (not listed, but pimelic below)	Dicarboxylic acid	0.27 [0.74]	0.49 [1.75]	0.361
Glycine	α-Amino acids	0.06 [0.44]	0.1 [0.31]	0.816
Homoserine	L-α-Amino acids	0.58 [1.4]	0.56 [1.25]	0.597
Isoleucine	Isoleucine and derivatives	0.46 [1.38]	0.35 [2.09]	0.756
Phenylalanine	Phenylalanine and derivatives	0.22 [1.14]	0.3 [2.09]	0.524
Proline	Proline and derivatives	0.54 [0.99]	0.45 [0.78]	0.847
Serine	Serine and derivatives	0.29 [1.2]	0.48 [1.46]	0.689
Threonine	L-α-Amino acids	0.21 [1.32]	0.34 [1.78]	0.432
Tyramine	Phenethylamines	0.19 [0.45]	0.36 [0.39]	0.148
Valine	Valine and derivatives	0.53 [1.5]	0.4 [1.7]	0.584
Allo-Inositol	Cyclitol	0.03 [0.06]	0.05 [0.08]	0.246
Ascorbic acid (Vitamin C)	Organic acid	0.08 [0.24]	0.07 [0.26]	0.756
5-Aminoimidazole-4-carboxamide	Aminoimidazoles	0.22 [0.29]	0.17 [0.24]	0.297
Pantothenic acid	β-Amino acids and derivatives	0.74 [0.88]	0.6 [1.15]	0.863
Arabitol (Arabitol = Arabinol)	Sugar alcohol	0.69 [0.87]	0.4 [1.11]	0.980
Benzene-1,2,4-triol (Hydroxyquinol)	Phenolic compound	0.32 [1.4]	0.39 [1.46]	0.675
(1R,2S)-cis-1,2-dihydro-1,2-naphthalenediol	Naphthalenes	0.62 [1.39]	0.35 [2.1]	0.771
cis-1,2-Cyclohexanediol	Cyclohexanediols	0.45 [1.53]	0.39 [0.76]	0.763
Dithiothreitol (DTT)	Thiol derivative of a sugar alcohol	1.08 [0.81]	0.98 [0.35]	0.486
Glycerol	Sugar alcohol	0.92 [1.4]	0.92 [1.77]	0.988
2-Butyne-1,4-diol	Diol	0.01 [0.01]	0.01 [0.01]	0.682
Ribitol (Adonitol)	Sugar alcohol	0.37 [0.76]	0.37 [1.21]	0.925
Sorbitol	Sugar alcohol	0.28 [0.86]	0.24 [1]	0.398
Threitol	Sugar alcohol	0.67 [0.78]	0.42 [0.85]	0.696
Acetol (Hydroxyacetone)	α-Hydroxy ketone	0.07 [0.27]	0.08 [0.63]	0.450
Allose	Monosaccharide	0.45 [0.57]	0.69 [1.02]	0.036
6-Deoxy-D-glucose	Monosaccharide	0.39 [1.47]	0.34 [1.61]	0.577
1,3-Dihydroxyacetone	Monosaccharide	0.39 [0.88]	0.34 [1.85]	0.925
Fructose	Monosaccharide	0.71 [0.47]	0.71 [1.39]	0.741
Glucopyranose	Monosaccharide	0 [0]	0 [0]	0.456
Glyceraldehyde	Monosaccharide	0.95 [1.38]	0.88 [1.83]	0.996
Glycerol 1-phosphate	Sugar alcohol phosphate	0.48 [0.7]	0.65 [0.72]	0.518
Glycerone phosphate	Ketose phosphate	0.02 [0.04]	0.06 [0.17]	0.025
Lactose	Disaccharide	0.34 [1.39]	0.11 [1.4]	0.611
Mannose	Monosaccharide	0.58 [0.99]	0.53 [1.32]	0.996
Sucrose	Disaccharide	0.15 [1.3]	0.57 [1.47]	0.550
Trehalose	Disaccharide	0.16 [0.29]	0.28 [0.49]	0.030
3-Phosphoglycerate	Sugar acids and derivatives	0.16 [0.63]	0.2 [1.1]	0.238
3-Phosphoglyceric acid	Organic acid phosphate	0.75 [0.81]	0.81 [1.27]	0.988
9-Fluorenone	Polycyclic aromatic ketone	0.25 [0.65]	0.31 [0.66]	0.653
2′-Deoxycytidine	Nucleoside	0.11 [0.14]	0.1 [0.09]	0.393
Thymine	Nucleobase	0.2 [1.5]	0.34 [2.05]	0.91
Alpha-Santonin	Sesquiterpene lactone	0.5 [1.75]	0.7 [1.31]	0.988
Benzoic acid	Aromatic carboxylic acid	0.9 [1.25]	0.63 [2.27]	0.756
1-Aminocyclopropane-1-carboxylic acid	α-Amino acids	0.59 [0.96]	0.49 [0.71]	0.832
Dehydroascorbic acid	Oxidized form of vitamin C	1.01 [0.91]	0.88 [0.74]	0.480
Galacturonic acid	Uronic acid	0.28 [1.78]	0.54 [1.42]	0.618
Glucoheptonic acid	Sugar acid	0.15 [1]	0.22 [0.76]	0.801
Gluconic acid	Aldonic acid	0.26 [0.23]	0.28 [0.23]	0.075
Gluconic acid lactone	Lactone form of gluconic acid	0.17 [0.77]	0.61 [1.27]	0.027
Glucosaminic acid	Amino sugar acid	0.26 [1.36]	0.68 [1.4]	0.316
Glucuronic acid	Uronic acid	0.58 [1.77]	0.47 [1.83]	0.832
Glyceric acid	Sugar acid	0.26 [1.55]	0.29 [2.33]	0.632
Glycolic acid	α-Hydroxy acid	0.12 [1.28]	0.08 [1.52]	0.557
Gulonic acid γ-lactone	Lactone of gulonic acid	0.51 [1.86]	0.47 [2.02]	0.625
Homogentisic acid	Aromatic acid	0.39 [0.61]	0.6 [1.12]	0.307
Itaconic acid	Dicarboxylic acid	1.05 [1.7]	0.51 [1.84]	0.356
Lactamide	Amide	0.49 [0.95]	0.43 [0.55]	0.689
Lactic acid	α-Hydroxy acid	0.09 [0.14]	0.15 [0.13]	0.242
Lactobionic acid	Fatty acyl glycosides of saccharides	0.23 [0.47]	0.21 [1.51]	0.711
Lactulose	Disaccharide	0.36 [0.83]	0.55 [0.81]	0.197
Lyxose	Monosaccharide	0.6 [1.35]	0.58 [2.52]	0.933
Maleamic acid	Unsaturated amide	0.44 [1.36]	0.44 [2.05]	0.917
Malonic acid	Dicarboxylic acid	0.45 [1.48]	0.35 [2.21]	0.957
Methyl-β-D-galactopyranoside	O-Glycosyl compounds	0.41 [0.97]	0.56 [1.02]	0.393
Methylmalonic acid	Dicarboxylic acids and derivatives	0.86 [1.29]	0.33 [1.49]	0.902
Neohesperidin	Flavonoid glycoside	0.05 [0.48]	0.06 [0.63]	0.878
O-Phosphocolamine (O-phosphoethanolamine)	Phosphoethanolamines	0.17 [0.73]	0.25 [1.74]	0.250
10-Hydroxydecanoic acid	Medium-chain hydroxy acids	0.47 [1.35]	0.64 [1.61]	0.597
Oxalic acid	Dicarboxylic acid	0.87 [1.11]	0.67 [2.34]	0.763
*p*-Cresol (4-methylphenol)	Phenolic compound	0.01 [0.02]	0 [0.02]	0.733
*p*-Hydroxyphenyllactic acid	Aromatic α-hydroxy acid	0.32 [1.44]	0.75 [2.2]	0.404
Paraoxon	Organophosphate compound	0.89 [0.99]	1.12 [0.54]	0.036
Pentonic acid	Sugar acids and derivatives	0.17 [0.32]	0.18 [0.3]	0.756
Phenyl-β-D-glucopyranoside	Phenolic glycosides	0.39 [0.92]	0.36 [1.97]	0.840
Phosphoric acid (orthophosphate)	Phosphorus oxoacid	0.25 [0.15]	0.26 [0.2]	0.382
Pi (inorganic phosphate)	Inorganic phosphate	0.01 [0.11]	0.01 [0.14]	0.456
Pimelic acid	Dicarboxylic acid	0.04 [2.39]	0.08 [2.57]	0.480
Pipecolic acid	L-α-Amino acids	0.27 [1.5]	0.38 [2.03]	0.794
Porphine	Porphyrin	0.41 [0.91]	0.45 [1.26]	0.722
Pyrogallol (1,2,3-benzenetriol)	Phenolic compound	0.37 [0.58]	0.4 [0.91]	0.087
Quinic acid	Cyclohexanecarboxylic acid	0.37 [1.19]	0.44 [1.35]	0.316
Ribonic acid γ-lactone	Lactone of ribonic acid	0.76 [1.15]	0.61 [1.3]	0.980
Sedoheptulose anhydride	Sugar derivative	0.13 [0.66]	0.12 [2.08]	0.809
Shikimic acid	Cyclohexene triol carboxylic acid	0.47 [0.47]	0.66 [0.49]	0.092
Sorbose	Monosaccharide	0.26 [0.8]	0.4 [0.54]	0.726
Sphingosine	Long-chain amino alcohol	0.06 [0.58]	0.04 [0.43]	0.894
Succinic acid	Dicarboxylic acid	0.29 [1.71]	0.35 [2.12]	0.618
Tartaric acid	Dihydroxy dicarboxylic acid	0.29 [0.62]	0.25 [1.08]	0.486
Tartronic acid	Hydroxy dicarboxylic acid	0.31 [1.24]	0.25 [1.93]	0.980
Threose	Monosaccharide	0.61 [1.68]	0.9 [1.75]	0.832
Trans-3-hexenedioic acid	Dicarboxylic acid	0.03 [0.47]	0.02 [0.08]	0.531
Urea	Ureas	0.2 [1.77]	0.21 [2.01]	0.972
3-Hydroxypropanoic acid	β-Hydroxy acid	1.03 [1.78]	0.89 [1.8]	0.275
Xanthine	Purine base	0.10 [0.37]	0.24 [0.92]	0.011
Cellobiose	Disaccharide	0.3 [1.31]	0.37 [1.35]	0.518
Fucose	Hexoses	0.35 [1.4]	0.34 [1.58]	0.771
Galactinol	O-Glycosyl compounds	0.36 [0.66]	0.41 [0.84]	0.427
Isopropyl β-D-thiogalactopyranoside (IPTG)	Thiogalactoside	0.02 [0.17]	0.04 [0.15]	0.356
Capric acid (Decanoic acid)	Fatty acid	0.86 [1.78]	0.41 [1.28]	0.511
Caprylic acid (Octanoic acid)	Fatty acid	0.56 [1.9]	0.07 [1.81]	0.794
Lauric acid (Dodecanoic acid)	Fatty acid	0.51 [1.15]	0.4 [0.97]	0.427
Methyl caprylate (Methyl octanoate)	Fatty acid ester	0 [0.02]	0 [0.05]	0.230
Myristic acid (Tetradecanoic)	Fatty acid	0.58 [1.67]	0.3 [1.79]	0.584
Palmitic acid (Hexadecanoic)	Fatty acid	0.44 [1.86]	0.48 [1.38]	0.832
Citramalic acid	Methylmalic acid	0.46 [2.03]	0.59 [1.51]	0.505
α-Ketoglutaric acid	Keto-dicarboxylic acid	0.63 [1.28]	0.46 [1.86]	0.878
Citric acid	Tricarboxylic acid	0.78 [0.65]	0.8 [0.95]	0.632
Erythrose-4-phosphate	Sugar phosphate	0.99 [0.54]	1.01 [0.33]	0.660
Isocitric acid	Tricarboxylic acid	0.13 [0.39]	0.32 [0.52]	0.040
Malic acid	Hydroxy dicarboxylic acid	0.65 [1.39]	0.49 [1.45]	0.972
Oxaloacetic acid	Keto-dicarboxylic acid	0.51 [0.7]	0.28 [0.91]	0.741
Linoleic acid	Fatty acid	0.31 [1.46]	0.45 [1.23]	0.996
Methyl linolenate (Methyl 18:3)	Fatty acid ester	0.45 [0.65]	0.43 [1.24]	0.611
Oleic acid	Fatty acid	0.29 [1.8]	0.44 [1.45]	0.847
Palmitoleic acid	Fatty acid	0.60 [0.92]	0.68 [1.14]	0.361
trans,trans-Farnesol	Sesquiterpene alcohol	0.75 [1.19]	0.58 [1.71]	0.557

The data are presented as the median [IQR]; GDM: gestational diabetes mellitus; MWU: Mann–Whitney U test.
